# SARCDNet-an enhanced deep learning network for change detection from bi-temporal SAR images

**DOI:** 10.1038/s41598-025-31488-y

**Published:** 2025-12-31

**Authors:** Vibha Damodara Kevala, Vishal Mukundan, Sravya Nedungatt, Shilpa Suresh, Shyam Lal

**Affiliations:** 1 Department of Electronics and Communication Engineering, National Institute of Technology Karnataka,Surathkal, Mangaluru, 575025 India; 2 Department of Mechatronics, Manipal Institute of Technology, Manipal Academy of Higher Education, Manipal, India

**Keywords:** Change detection, Deep learning, SAR, Remote sensing, Climate sciences, Environmental sciences, Natural hazards

## Abstract

Change detection analysis is a crucial process in microwave remote sensing enabling the identification of changes on the Earth’s surface due to natural and human activities. Bi-temporal Synthetic Aperture Radar (SAR) imaging is particularly effective for flood detection, environmental monitoring, disaster response and urban planning due to its all-weather, day-and-night capabilities. Conventional optical imaging methods face challenges such as cloud cover and daylight dependency making SAR a preferred alternative. The availability of SAR data from satellite resources such as RADARSAT, Sentinel-1, Envisat has helped in advancing change detection analysis. This paper introduces SARCDNet (SAR Change Detection Network) model which is an enhanced deep learning network for change detection from bi-temporal SAR images. Adaptive fusion block is designed, which incorporates the adaptive global filtering operation that extracts features in frequency domain and channel attention mechanism to enhance the relevance of extracted features. SARCDNet is computationally efficient design that mitigates the effects of speckle noise and improves the prediction accuracy. SARCDNet takes advantage of spatial and frequency domain methods and has demonstrated improved performance across public datasets namely Yellow river and Farmland which has higher incidence of speckle noise. The proposed SARCDNet model has demonstrated considerable performance improvement of 4.26% F1 score, 0.841% PCC, 4.92% $\kappa$ and 4.67% MCC in flood change detection in Chao Lake dataset.

## Introduction

The climate change due to man-made reasons have increasingly stressed the various resources available on earth whether its the forest, glaciers, sea ice or the very agricultural practices that sustain human life. The effects of climate change have been increasing whether its in the form of flood, droughts, glacier and sea ice melting. It is imperative to monitor these changes thus helping in assessing the potential impacts and subsequent disaster response arising due to these. Change detection analysis is a critical process used to identify the differences in spatial representation between the image taken before and after a time period or the occurrence of an event such as flood. The data used for the change detection analysis can be obtained using satellites, UAVs or aircraft. The importance of satellite based monitoring is increasing due to easier availability and its advantages such as higher resolution, coverage etc. Optical imaging is a commonly employed remote sensing technique that records visible and near-infrared radiation reflected from the Earth’s surface. As it takes images in the visible region, its functionality is similar to how humans perceive things making optical imagery easier to interpret. However optical imaging often encounters considerable challenges in change detection analysis, it’s reliance on daylight and clear weather conditions, its interpretability is often hampered due to clouds, fog and darkness. Consequently obtaining consistent time-series imagery for monitoring changes becomes problematic which often results in data deficiencies and compromised analysis. Synthetic Aperture Radar (SAR) is an active sensor based imaging technique which provides a powerful alternative to optical images. Instead of depending on the visible light, a SAR instrument generates its own pulses of microwave energy and transmits them towards the ground and precisely records the signal that is scattered back from the surface. This active mechanism makes SAR independent of solar illumination and atmospheric conditions which gives it an all-weather, 24/7 operational capability that ensures consistent and reliable data acquisition. Furthermore SAR imaging has higher temporal resolution resulting in faster information collection. A significant challenge inherent to SAR imaging is the presence of speckle noise. This phenomenon manifests as a granular, ”salt-and-pepper” texture throughout the image which is a direct consequence of the coherent nature of radar imaging. Speckle arises from the constructive and destructive interference of the radar waves returning from multiple and varied scattering elements contained within a single resolution cell on the ground. While various filtering algorithms are applied to reduce speckle and improve image interpretability they can sometimes have the unintended consequence of blurring fine details and sharp edges. Researchers have also explored the fusion of optical and SAR data to make better use of information from optical and SAR images^1–3^. The change detection analysis of SAR images has come from a long way from statistical techniques to traditional machine learning approaches and then finally to modern deep learning techniques. Rignot et al.^4^ proposed and compared methods for finding temporal changes in the ERS-1 SAR imagery and demonstrated that intensity ratioing suits multi-look intensity data while estimates of speckle’s temporal decorrelation serve as the basis for change detection which is found to be optimal for one-look complex or low-look intensity data, thereby highlighting their complementary roles in monitoring surface changes. White^5^ proposed a method for speckle noise reduction through neural networks. Dekker^6^ proposed a SAR image change detection method that filters the logarithmic-scaled ratio of images, which converts multiplicative speckle noise to additive noise for simpler and optimized filtering. Dai et al.^7^ proposed a change detection technique based on artificial neural network. Xiong et al.^8^ proposed a likelihood ratio based change detection method taking into account statistical distribution of SAR intensity images. Zheng et al.^9^ proposed a unique technique combining k-means clustering with difference image in order to get better change detection. Many techniques have been developed utilizing a hybrid approach involving an unsupervised clustering followed by supervised fine tuning. Gong et al.^10^ proposed a deep neural network for change detection which involves an unsupervised clustering on a difference image followed by a neural network to classify the pixels as changed or unchanged. Geng et al.^11^ proposed a technique which utilized constrictive autoencoder trained to learn the changed pixels from a log ratio difference images and bitemporal SAR images. Many techniques involving frequency domain have also been developed to take advantage of frequency domain properties for enhanced change detection. Gao et al.^12^ employed a convolutional wavelet neural network for sea ice change detection. Ma et al.^13^ proposed a attention based spatial-frequency approach for change detection of SAR images. Xie et al.^14^ proposed an attention-based spatial-frequency approach for change detection of SAR images called WBANet. This network incorporates a Wavelet-based Self-attention Module (WSM) that utilizes the Discrete Wavelet Transform (DWT) and its inverse (IDWT) for a lossless and multi-scale downsampling strategy within the self-attention mechanism which effectively preserves high-frequency information. The architecture also integrates a Bi-dimensional Aggregation Module (BAM) to enhance feature representation by capturing both spatial and channel features. Zhang et al.^15^ proposed CAMixer which features a Parallel Convolution and Attention Module (PCAM) and a Gated Feed-Forward Network (GFFN). The PCAM extracts local and global features by fusing shift convolution with self-attention. Concurrently, the GFFN enhances nonlinear feature transformation by employing multiscale depth-wise convolutions within a gating mechanism to emphasize significant features. Qu et al.^16^ proposed DDNet, a network for SAR change detection that effectively fuses spatial and frequency domain features. It employs the Discrete Cosine Transform (DCT) to extract frequency information which helps mitigate speckle noise while a Multi-Region Convolution (MRC) module adaptively captures local and contextual spatial details by prioritizing the central region of image patches. Kevala et al.^17^ proposed an enhancement to the DDNet architecture by incorporating an Atrous Spatial Pyramid Pooling (ASPP) block into the spatial domain branch. In this model the ASPP module processes the output from the Multi-Region Convolution (MRC) block to extract multi-scale features. These enhanced spatial features are then concatenated with the frequency-domain features from the Discrete Cosine Transform (DCT) branch to form a final comprehensive feature set for classification. Meng et al.^18^ proposed LANTNet which is a specialized architecture for change detection employing a convolutional stem to extract a hierarchical feature set from input data. The architecture’s key innovation is a layer attention module that explicitly models and leverages the interdependencies between features from different layers. This module dynamically weights the contribution of each layer’s features thereby creating a refined representation that captures the correlation between them to generate the final change map. The Siamese model architecture is yet another approach implemented by researchers in change detection. Weight sharing occurs during feature extraction in such an architecture. A vision transformer-based method for flood detection using SAR images has been proposed by Saleh et al.^19^. A Siamese model architecture for flood detection from SAR images was proposed by Tahermanesh et al.^20^. The reviewed literature highlights a significant evolution in SAR image change detection, moving from foundational statistical methods and early machine learning applications to sophisticated deep learning architectures incorporating techniques like unsupervised pre-training, autoencoders, and frequency domain analysis. The proposed model aims to mitigate the effects of speckle noise and enhance the relevance of extracted features using Adaptive Fusion Block thereby improving the classification results. The key contribution of this paper are as follows:

1. Adaptive Fusion Block (AFB) incorporates the channel attention mechanism in spatial domain and the global filter operation in frequency domain. Unlike DDNet’s fixed DCT features, AFB incorporates trainable weights that adaptively learns the frequency domain features. Furthermore, feature modulation is carried out by combining spatial and frequency domain features. Hence it mitigates the effects of speckle noise.
2. The proposed SARCDNet model is developed by combining AFB with a shallow CNN-based network. When compared to current state-of-the-art models, the AFB’s use of separable convolution significantly reduces the model complexity. As a result, SARCDNet requires fewer resources while achieving better classification results.

## Proposed model

**Fig. 1:**
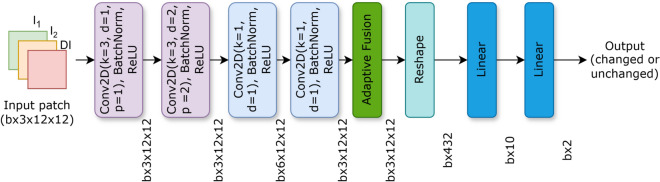
Proposed SARCDNet model architecture.

The pre ($I_{1}$) and post ($I_{2}$) event SAR images are first pre-processed by applying SRAD filter^21^ in order to enhance contrast and reduce speckle noise. Then a log-ratio difference image is generated. This technique emphasizes relative intensity differences which makes it highly effective for detecting subtle changes. Following the pre-processing step an unsupervised clustering technique is used to segment the data. This involves a two-phase approach starting with a binary Fuzzy C-Means (FCM) clustering^22^ which assigns each pixel a soft membership score for the ”changed” and ”unchanged” classes. Based on the distribution of these scores a more detailed multi-class FCM is applied which divides the image into five clusters. These clusters are sorted by their average intensity assuming higher intensities correlate with a higher likelihood of change. This initial clustering allows us to partition the image pixels into three distinct groups using a hierarchical strategy. At the end of this, we have three clusters namely changed ($S_c$), uncertain ($S_i$) and unchanged ($S_u$). With these pixel sets established, the training dataset for our proposed model is constructed by selecting 10% of the patches from the reliable categories which are cluster $S_c$ and $S_u$. We ensure an equal number of positive samples from $S_c$ and negative samples from $S_u$ to create a balanced training dataset. The input for our network is generated by extracting corresponding $t \times t$ image patches from the two temporal images $I_1$ and $I_2$ and stacking them to yield a two-channel tensor with dimensions $2 \times t \times t$. The proposed model is trained on this data and it is used to process the patches associated with the uncertain pixels in $S_i$ to determine their final classification and then generate the final binary change map. The proposed model SARCDNet consists of a series of convolutional layers followed by the adaptive fusion block and finally a dense layer to give the final output. The input is first passed through convolutional layer with kernel size of ${3 \times 3}$ with a dilation rate of 1. After this it again passes through a convolutional layer having a dilation rate of 2 this dilation helps in extraction of multiscale feature which is helpful in improving the performance of the network. Then the output from the above ${ 3 \times 3}$ convolutional layer is passed through a series of two ${1 \times 1}$ convolutional layer. The output of the above is then passed through the adaptive fusion block. The adaptive fusion block’s multi-scale feature extraction ensures that diverse change events whether small or large are effectively identified by reducing misclassification. By incorporating frequency-domain processing the block allows for more precise discrimination based on subtle shifts in surface textural and structural properties which is crucial for accurate binary labeling when changes are not just intensity-based. This coupled with its potential for speckle noise resilience helps prevent misclassifications due to sensor artifacts thereby minimizing both false positives and false negatives. The channel attention mechanism facilitates adaptive decision-making by enabling the model to dynamically focus on the most discriminative features signaling change which leads to a robust binary classifications across various scenarios. The output of the adaptive fusion block is passed through a linear layer which outputs either ”changed” or ”unchanged” in the form of 0 and 1 respectively. The proposed model’s architecture is given in Fig. 1.

### Adaptive fusion block

**Fig. 2:**
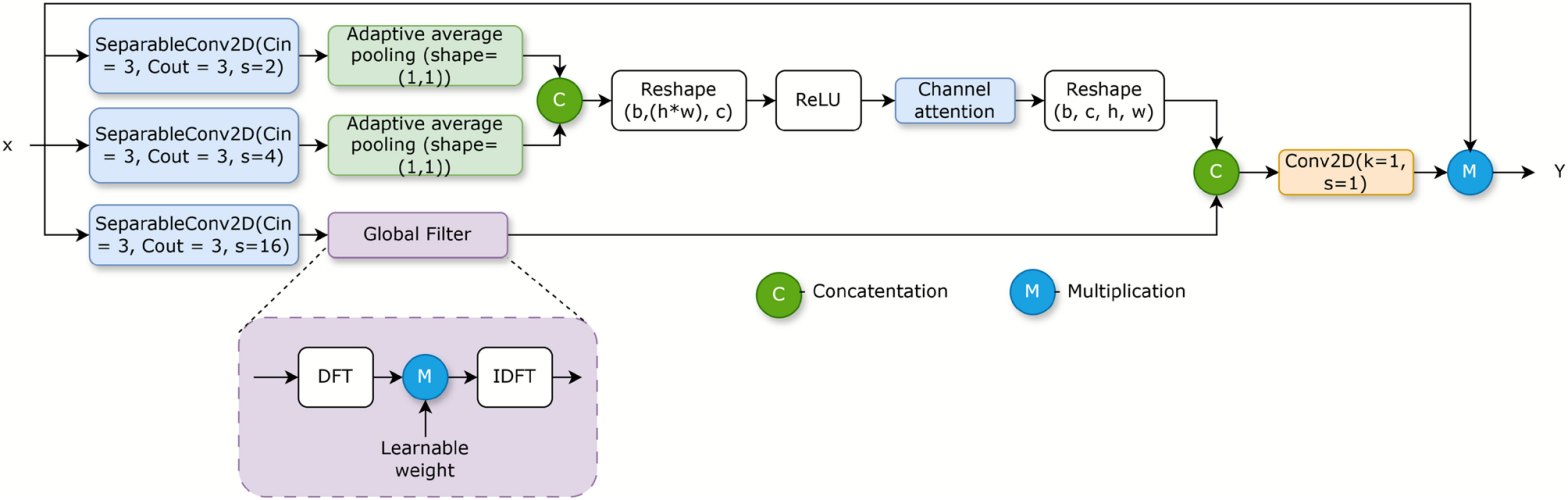
Adaptive fusion block.

The adaptive fusion block is designed to enhance feature representations for robust binary change detection particularly within challenging SAR imagery by synergistically integrating spatial and frequency domain information. The block is illustrated by Fig. 2. The architecture initiates parallel pathways for feature extraction. Firstly to capture changes across diverse spatial extents inherent in SAR scenes, multi-scale spatial features are extracted using computationally efficient depthwise separable convolutions with varied strides which is followed by adaptive average pooling on 1 st and 2nd branches to abstract these into global channel-wise descriptors. Concurrently a 3rd pathway processes features from a coarsely resolved spatial branch within the frequency domain using a GlobalFilter module^23^. GlobalFilter module employs a Fast Fourier Transform (FFT) and applies learnable complex-valued weights in the spectral domain and subsequently performs an inverse FFT to yield a global feature descriptor spatially reduced to ${1 \times 1}$. Such frequency-domain analysis is particularly beneficial for SAR data which enables the model to discern textural variations, periodic structures which potentially offers enhanced robustness to speckle noise by learning to emphasize specific frequency components.

To integrate the information derived from 1 st and 2nd parallel streams, a channel attention^24^ mechanism is employed to adaptively weigh the global descriptors from the different spatial scales thereby allowing the network to prioritize the most salient scale-specific information for change detection. The resulting attention-weighted spatial context is then concatenated with the global features obtained from the frequency-domain pathway. This composite feature vector which is rich in multi-scale spatial and frequency-domain features is further refined and its dimensionality adjusted via a ${1 \times 1}$ convolutional layer, producing a comprehensive global context vector. The critical final stage of the adaptive fusion block involves the element-wise multiplication of this derived global context vector with the original input feature map. This operation acts as an adaptive modulation which dynamically scales the input features to amplify change-relevant signals consistent with the learned global context while suppressing irrelevant information and noise. The equations describing the operation of this block is given in equations (1 - 7).

To incorporate diverse receptive fields and frequency information we designed three parallel feature extraction arms which is described by equations 1-3. Arm 1 uses a $3 \times 3$ convolution with stride 2 followed by global average pooling(GAP), it is described as follows.

1 $$\begin{aligned} C_1 = GAP(\textrm{Conv}_{3 \times 3}({\textbf {x}})) \end{aligned}$$

Arm 2 applies a $3 \times 3$ convolution with stride 4 followed by GAP, it is described as follows.

2 $$\begin{aligned} C_2 = GAP(\textrm{Conv}_{3 \times 3}({\textbf {x}})) \end{aligned}$$

Arm 3 operates in the frequency domain using a learnable filter.

3 $$\begin{aligned} C_3 = f^{-1}[K \odot f[\textrm{Conv}_{3 \times 3}({\textbf {x}})]] \end{aligned}$$

where $f[\cdot ]$ and $f^{-1}[\cdot ]$ describes the 2D Fast Fourier Transform (FFT) and its inverse (IFFT) respectively and K is a learnable frequency domain filter.

4 $$\begin{aligned} C_{11} = \textrm{Concat}(C_1, C_2) \end{aligned}$$

Multi-head attention is applied on the concatenated output to capture global contextual dependencies. Number of heads used is 4. The following equation describes the operation.

5 $$\begin{aligned} C_{12} = \textrm{MultiHeadAttention}(C_{11}) \end{aligned}$$

The output of the attention module is concatenated with the frequency-domain features from Arm 3, its described as follows.

6 $$\begin{aligned} C_f = \textrm{Concat}(C_{12}, C_3) \end{aligned}$$

Finally, the fused feature representation $C_f$ is projected via a $1 \times 1$ convolution and the original input x modulated through element-wise multiplication to produce the final output of AFB:

7 $$\begin{aligned} {\textbf {Y}} = \textrm{Conv}_{1 \times 1}(C_f) \odot {\textbf {x}} \end{aligned}$$

## Training and evaluation metrics

### Dataset

This study used five datasets namely Ottawa, Yellow river, Farmland, Sulzberger and Chao Lake. Ottawa dataset consists of image pair taken with almost a year gap in Ottawa City in Canada in May and August 1997 along with ground truth. The image is captured by RADARSAT SAR with size of image being $\ 290 \times 350$ pixels. Sulzberger dataset consists of SAR image pairs of Sulzberger Ice Shelf located in Antarctica along with changed ground truth. The SAR image is taken by Envisat satellite with size being $\ 256 \times 256$ pixels. The image is taken after the breaking of ice shelf after the tsunami in March 2011. We have considered one image pair from the Yellow river dataset (which actually consists of three image pairs) along the Yellow river Estuary in China with ground truth. The image is taken using RADARSAT 2 satellite with image size of $\ 291 \times 306$ pixels, the image is taken on June 2008 and June 2009. Captured by the Sentinel-1 sensor in May 2020, the Chao Lake I dataset provides imagery of the Chao lake area in China during its record high water level. The image size is $\ 384 \times 384$. The Farmland dataset was taken in June 2008 and June 2009 using RADARSAT from Yellow river Estuary in China. The image size is $\ 306 \times 291$. For each of the five image pairs the pre-classification is performed to generate pseudolabels. Then SARCDNet is trained separately for each image pair.

### Training setup

The training is done on P100 GPU with 16GB of memory in kaggle. The Adam optimizer with an initial learning rate of 0.0001 and other default parameters is used. SARCDNet is trained for 50 epochs with a batch size of 128. The loss function employed is categorical cross entropy. It took 70 s on an average to train for each image pair.

### Evaluation metrics

The proposed model and the state-of-the-art models are compared using F1 score, Kappa coefficient ($\kappa$), Matthews correlation coefficient(MCC) and percentage of correct classification(PCC). F1 score in simple terms is the harmonic mean of precision and recall. $\kappa$ is basically a statistical measure that quantify the level of agreement between two observers. MCC is a measure of quality in a binary classification task, which spans from −1 to +1. PCC is the ratio of correctly classified pixel to the total amount of pixels present. Equations 8 to 12 describe the evaluation metrics used.

8 $$\begin{aligned} \text {PCC}={(\text {TP}+\text {TN})}/{(\text {TP}+\text {FP}+\text {TN}+\text {FN})} \end{aligned}$$

9 $$\begin{aligned} \kappa =\frac{\text {PCC}-\text {PRE}}{1-\text {PRE}} \end{aligned}$$

where,

10 $$\begin{aligned} \text {PRE}=\frac{(\text {TP}+\text {FP})\cdot \text {Pc}+(\text {FN}+\text {TN})\cdot \text {Pu}}{(\text {TP}+\text {FP}+\text {FN}+\text {TN})^{2}} \end{aligned}$$

where, Pu and Pc are the actual number of unchanged and changed pixels in ground truth.

11 $$\begin{aligned} F1\ score = (2*\text {TP})/(2*\text {TP}+\text {FP}+\text {FN}) \end{aligned}$$

12 $$\begin{aligned} \text {MCC} = \frac{(\text {TP}*\text {TN} - \text {FP}*\text {FN})}{\sqrt{((\text {TP}+\text {FP})*(\text {TP} + \text {FN})*(\text {TN} + \text {FP})*(\text {TN} + \text {FN}))}} \end{aligned}$$

## Results

### Analysis of patch size

**Fig. 3:**
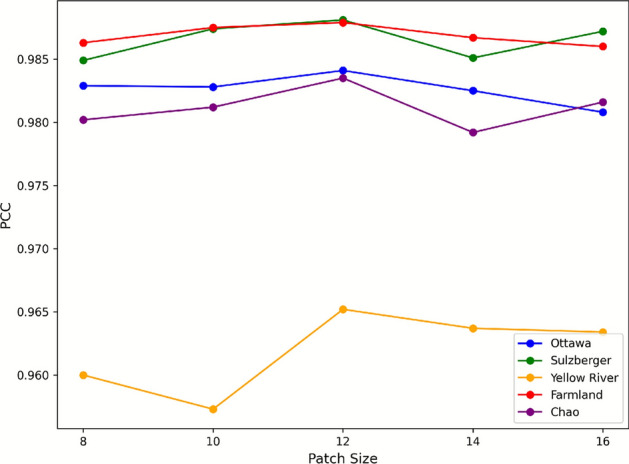
Patch size analysis.

In order to choose the best patch size so that better spatial contextual information is captured, the model is trained for different patch sizes. The patch sizes choosen for this study are 8,10,12,14 and 16. From the graph in Fig. 3, we can observe that the PCC value increases at first and then peaks at patch size 12 and then again goes down. The selection of patch size is important as it should be balanced in such a way that sufficient spatial context is preserved but computational burden is also less. Hence patch size of 12 is used for the proposed model’s evaluation.

### Ablation study

The proposed model SARCDNet employs effective utilization of adaptive fusion block (AFB). Three intermediate models namely W/O AFB (no AFB block), W/O attention (no attention applied in AFB and direct concatenation with output of global filter), W/O global filter (no global filter operation in AFB and direct concatenation of channel attention output with separable convolution output) are compared with the model that includes AFB. The ablation study results are shown in Table 1. On the Ottawa dataset the inclusion of the AFB in the proposed model resulted in improvements across all metrics. The PCC increased by 0.09%, $\kappa$ by 0.37%, F1 Score by 0.32% and MCC by 0.43%. For the Sulzberger dataset, the impact of the AFB was even more pronounced. The proposed model achieved a 0.54% increase in PCC, $\kappa$ improved by 1.72%, F1 Score by 1.36%, and MCC by 1.69%. Similarly for the Yellow river dataset the proposed model resulted in a 0.57% increase in PCC when compared to W/O AFB intermediate model. The $\kappa$ increased by 1.88%, the F1 Score by 1.47%, and the MCC by 1.97%. On the Farmland dataset PCC rose by 0.16%, $\kappa$ by 1.82%, F1 Score by 1.36%, and MCC by 1.48%. The proposed model achieved a 1.61% increase in PCC. The $\kappa$ saw a considerable jump of 9.42%. Similarly, the F1 Score improved by 8.25%, and the MCC by 8.61% on the Chao dataset. The ablation study results of the W/O attention and W/O global filter shows the significance of the channel attention and the global filter operations in the AFB in enhancing the feature representation and discrimination while handling the complexities in the datasets.

**Table 1:** Ablation study results.

Dataset	Model	PCC	$\kappa$	F1 score	MCC
Ottawa	W/O AFB	0.9832	0.9366	0.946	0.936
	W/O attention	0.9838	0.9389	**0.949**	0.939
	W/O global filter	0.9836	0.9386	0.948	0.939
	With AFB (SARCDNet)	**0.9841**	**0.9401**	**0.949**	**0.940**
Sulzberger	W/O AFB	0.9828	0.9455	0.956	0.945
	W/O attention	0.9839	0.9492	0.959	0.950
	W/O global filter	0.983	0.9461	0.957	0.946
	With AFB (SARCDNet)	**0.9881**	**0.9618**	**0.969**	**0.961**
Yellow river	W/O AFB	0.9597	0.8631	0.887	0.863
	W/O attention	0.9626	0.8708	0.893	0.871
	W/O global filter	0.9607	0.8647	0.889	0.865
	With AFB (SARCDNet)	**0.9652**	**0.8793**	**0.900**	**0.88**
Farmland	W/O AFB	0.9863	0.8731	0.883	0.876
	W/O attention	0.9855	0.8712	0.879	0.871
	W/O global filter	0.9859	0.8731	0.881	0.873
	With AFB (SARCDNet)	**0.9879**	**0.889**	**0.895**	**0.889**
Chao	W/O AFB	0.9679	0.8192	0.836	0.825
	W/O attention	0.9711	0.8325	0.848	0.836
	W/O global filter	0.9752	0.8511	0.865	0.852
	With AFB (SARCDNet)	**0.9835**	**0.8964**	**0.905**	**0.896**

**Table 2:** Results on Ottawa, Sulzberger and Yellow river dataset.

Models	Ottawa				Sulzberger				Yellow river			
	PCC	$\kappa$	F1 Score	MCC	PCC	$\kappa$	F1 Score	MCC	PCC	$\kappa$	F1 Score	MCC
DDNet (2021)	98.17	93.06	0.941	0.930	98.61	95.58	0.964	0.955	95.66	84.79	0.873	0.849
LANTNet (2022)	98.30	93.50	0.945	0.935	98.78	96.10	0.968	0.961	95.21	83.01	0.858	0.832
CAMixer (2023)	98.13	92.83	0.939	0.928	98.75	95.96	0.967	0.959	95.01	82.26	0.852	0.825
MDDNet (2024)	98.30	93.52	0.945	0.935	98.56	95.36	0.962	0.953	95.17	82.99	0.858	0.831
WBANet (2024)	98.22	93.17	0.942	0.932	98.36	94.75	0.957	0.947	95.70	85.08	0.876	0.851
**SARCDNet**	**98.41**	**94.01**	**0.949**	**0.940**	**98.81**	**96.18**	**0.969**	**0.961**	**96.52**	**87.93**	**0.900**	**0.880**

**Table 3:** Results on Farmland and Chao dataset.

Models	Farmland				Chao			
	PCC	$\kappa$	F1 Score	MCC	PCC	$\kappa$	F1 Score	MCC
DDNet (2021)	98.47	85.93	0.867	0.859	97.29	84.45	0.859	0.849
LANTNet (2022)	98.62	86.55	0.872	0.869	97.53	85.44	0.868	0.856
CAMixer (2023)	98.70	87.34	0.880	0.877	96.89	81.95	0.836	0.823
MDDNet (2024)	98.58	85.96	0.867	0.865	97.51	85.07	0.864	0.851
WBANet (2024)	98.65	87.43	0.881	0.874	97.19	83.67	0.852	0.840
**SARCDNet**	**98.79**	**88.90**	**0.895**	**0.889**	**98.35**	**89.64**	**0.905**	**0.896**

### Performance comparison with benchmark models

**Fig. 4:**
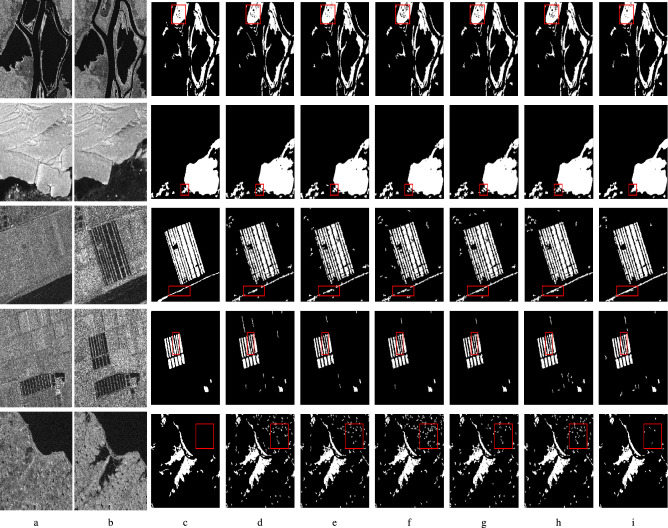
Visualisation of results. First row: Ottawa dataset, second row: Sulzberger dataset, third row: Yellow river dataset, fourth row: Farmland dataset, fifth row: Chao dataset. (**a**) Pre, (**b**) Post, (**c**) Ground Truth, (**d**) DDNet results, (**e**) LANTNet results, (**f**) CAMixer results, (**g**) MDDNet results, (**h**) WBANet results, (**i**) Proposed Model results.

The proposed SARCDNet model is compared with five state-of-the-art SAR change detection deep learning models namely DDNet, LANTNet, CAMixer, MDDNet, WBANet. The numerical metrics computed are tabulated in Table 2 and Table 3. On the Ottawa dataset the proposed model resulted the highest PCC value with 98.41%, the highest $\kappa$ with 94.01%, the highest F1 Score with 0.949 and the highest MCC with 0.940. When compared to the best performing reference model which is the MDDNet, the proposed model shows notable improvements that translates to an increase of approximately 0.423% in F1 Score, an increase of about 0.112% in PCC, an increase of about 0.524% in $\kappa$ and an increase of about 0.535% in MCC. The results obtained on the Sulzberger dataset showcased best metrics by SARCDNet with respect to PCC, $\kappa$ and F1 score. When compared to the best performing reference model LANTNet, the proposed SARCDNet demonstrates incremental improvements. Specifically, SARCDNet shows an increase of approximately 0.103% in F1 Score, 0.030% in PCC, and 0.083% in $\kappa$ over LANTNet. The MCC score for the proposed SARCDNet model is 0.961 which is equivalent to LANTNet’s MCC. The result obtained on the Yellow river dataset shows best results from proposed SARCDNet. The proposed SARCDNet surpasses the next best performing WBANet model with a substantial increase of approximately 2.74% in F1 Score, about 0.857% in PCC, a 3.35% increase in $\kappa$, and a 3.41% increase in MCC which collectively signifies a robust improvement in classification accuracy and reliability on this challenging dataset. The performance here underscores that the proposed model is effective in mitigating the effect of speckle noise through the use of AFB. The result on the Farmland dataset shows best performance by the proposed SARCDNet model. The proposed model’s F1 Score of 0.895 represents an increase of approximately 1.59% over WBANet. Its PCC of 98.79% is an increase of about 0.091% over CAMixer. The $\kappa$ of 88.90% marks an increase of approximately 1.68% over WBANet and the MCC of 0.889 is an increase of about 1.37% over CAMixer. The proposed SARCDNet model once again proves that its effective in reducing the effects of speckle noise through its spatial and frequency attention techniques employed in the AFB. The result obtained on the Chao dataset records the highest PCC of 98.35%, $\kappa$ of 89.64%, F1 score of 0.905 and MCC of 0.896 by the proposed SARCDNet. The proposed model’s F1 score indicates substantial increase of approximately 4.26%, PCC shows an increase of about 0.841%, $\kappa$ with an increase of about 4.92% and its MCC with an increase of about 4.67% when compared to next best performing reference model LANTNet. Overall, SARCDNet showcased substantial improvements in the change detection performance with respect to Yellow river and Chao lake datasets. Fig. 4 shows the change detection results of SARCDNet model and reference models considered for comparison. Some noteworthy regions in the image are highlighted with red box. With respect to Ottawa dataset, the ground truth (row 1, column c) has very few unchanged pixels. Whereas in the reference model predictions (row 1, columns d-h) there are many unchanged pixels when compared to SARCDNet prediction (row 1, column i). Similar observation is made with respect to Sulzberger dataset also (row 2, columns c-i). This indicates that with respect to Ottawa and Sulzberger datasets SARCDNet has effectively reduced false negatives. With respect to Yellow river and Farmland datasets, close observation of the red highlighted region shows that the edges are well preserved in the SARCDNet predicted change map when compared to the ground truth. MDDNet and WBANet (rows 3–4, columns g-h) also showcase good edge preservation but they also exhibit overall higher false positives (white grainy pixels). With respect to Chao dataset, SARCDNet (row 5, column i) shows best prediction with very less false positives than all other reference models (row 5, columns d-h) considered for comparison.

Table 4 presents the FLOPs and the total trainable parameters of SARCDNet and the reference models considered for comparison. SARCDNet has lowest trainable parameters that indicates lower training time requirement to train SARCDNet. Also, since the number of FLOPs is considerably low indicating that SARCDNet has lower resource requirement also. It shows that SARCDNet is practically significant for change detection tasks.

**Table 4:** Comparison of computational complexity.

Model	FLOPs (K)	Parameters (K)
DDNet (2021)	114.86	6.71
LANTNet (2022)	1210	24.96
CAMixer (2023)	333.86	34.21
MDDNet (2024)	350000	7098
WBANet (2024)	23.55	7.16
**SARCDNet**	47.16	6.287

## Conclusion

This paper proposed SARCDNet model for change detection from bi-temporal SAR images. The proposed SARCDNet model addressed the inherent challenges of change detection through its innovative adaptive fusion block. The AFB’s adaptive global filter operation efficiently extracted frequency-domain characteristics, considerably improving border delineation and reducing speckle noise. This was further proven by its performance on the Yellow river and Farmland datasets, which had more speckle noise. The AFB’s channel attention enhanced the relevance of extracted features in the spatial domain. Furthermore, the SARCDNet model demonstrated its capability in mitigating false positives which was clearly observed in the results from the Chao Lake and Ottawa datasets. When compared to the best reference models, the proposed SARCDNet model outperforms them quantitatively on five different datasets. The datasets analyzed in this study have similar dimensions. The use of 10% of the changed and unchanged class was nearly same in all five scenarios. The model’s performance on a larger image will be explored in future work. This will make it possible to ascertain whether the model’s performance is being constrained by the size of the dataset. To improve outcomes, alternative methods such as Gauss log-ratio and wavelet fusion could be utilized in place of the existing log-ratio approach.

## Data Availability

The publicly available SAR change detection dataset can be accessed from https://github.com/summitgao/SAR_changed_Detection_Data12. The python code of SARCDNet model is available from the corresponding author on reasonable request.

## References

[1] Naanjam, R. & Ahmadi, F. F. An improved self-training network for building and road extraction in urban areas by integrating optical and radar remotely sensed data. *Earth Sci. Informatics 2024 17:3* **17**, 2159–2176. 10.1007/S12145-024-01270-1 (2024).

[2] Ahmadi, F. F., Naanjam, R. & Salimi, A. Developing an automatic training technique based on integration of radar and optical remotely sensed images for building extraction. *Earth Sci. Informatics 2023 17:1* **17**, 131–143. 10.1007/S12145-023-01154-W (2023).

[3] Farhadi, H., Kiani, A., Ebadi, H. & Asgary, A. Development of an automatic time-series flood mapping framework using sentinel-1 and 2 imagery. *Stoch. Environ. Res. Risk Assess. 2025 39:6* **39**, 2627–2655. 10.1007/S00477-025-02987-1 (2025).

[4] Rignot, E. & van Zyl, J. yChange detection techniques for ers-1 sar data. *IEEE Transactions on Geosci. Remote. Sens.* **31**, 896–906. 10.1109/36.239913 (1993).

[5] WHITE, R. G. Change detection in sar imagery. *Int. J. Remote. Sens.* **12**, 339–360. 10.1080/01431169108929656 (1991).

[6] Dekker, R. J. Speckle filtering in satellite sar change detection imagery. *Int. J. Remote. Sens.* **19**, 1133–1146. 10.1080/014311698215649 (1998).

[7] Dai, X. L. & Khorram, S. Remotely sensed change detection based on artificial neural networks. *Photogramm. engineering remote sensing* **65**, 1187–1194 (1999).

[8] Xiong, B., Chen, J. M. & G. K. A change detection measure based on a likelihood ratio and statistical properties of sar intensity images. *Remote. Sens. Lett.* **3**, 267–275. 10.1080/01431161.2011.572093 (2012).

[9] Zheng, Y., Zhang, X., Hou, B. & Liu, G. Using combined difference image and *k*-means clustering for sar image change detection. *IEEE Geosci. Remote. Sens. Lett.* **11**, 691–695. 10.1109/LGRS.2013.2275738 (2014).

[10] Gong, M., Zhao, J., Liu, J., Miao, Q. & Jiao, L. Change detection in synthetic aperture radar images based on deep neural networks. *IEEE Transactions on Neural Networks Learn. Syst.* **27**, 125–138. 10.1109/TNNLS.2015.2435783 (2016). 10.1109/TNNLS.2015.2435783 26068879

[11] Geng, J., Wang, H., Fan, J. & Ma, X. Change detection of sar images based on supervised contractive autoencoders and fuzzy clustering. In *2017 International Workshop on Remote Sensing with Intelligent Processing (RSIP)*, 1–3. 10.1109/RSIP.2017.7958819 (2017).

[12] Gao, F., Wang, X., Gao, Y., Dong, J. & Wang, S. Sea ice change detection in sar images based on convolutional-wavelet neural networks. *IEEE Geosci. Remote. Sens. Lett.* **16**, 1240–1244. 10.1109/LGRS.2019.2895656 (2019).

[13] Ma, L., Wang, L., Zhao, C., E, J. & Ohtsuki, T. Multilayer attention mechanism for change detection in sar image spatial-frequency domain. In *2023 IEEE International Conference on Image Processing (ICIP)*, 2110–2114. 10.1109/ICIP49359.2023.10222565 (2023).

[14] Xie, J., Gao, F., Zhou, X. & Dong, J. Wavelet-based bi-dimensional aggregation network for sar image change detection. *IEEE Geosci. Remote. Sens. Lett.* **21**, 1–5. 10.1109/LGRS.2024.3431532 (2024).

[15] Zhang, H. et al. Convolution and attention mixer for synthetic aperture radar image change detection. *IEEE Geosci. Remote. Sens. Lett.* **20**, 1–5. 10.1109/LGRS.2023.3318593 (2023).

[16] Qu, X., Gao, F., Dong, J., Du, Q. & Li, H.-C. Change detection in synthetic aperture radar images using a dual-domain network. *IEEE Geosci. Remote. Sens. Lett.* **19**, 1–5. 10.1109/LGRS.2021.3073900 (2022).

[17] Kevala, V.D., Ravi, S., N, S. K. B. & Lal, S. Modified dual domain network for sar change detection. In *2024 IEEE International Conference on Electronics, Computing and Communication Technologies (CONECCT)*, 1–4. 10.1109/CONECCT62155.2024.10677077 (2024).

[18] Meng, D., Gao, F., Dong, J., Du, Q. & Li, H.-C. Synthetic aperture radar image change detection via layer attention-based noise-tolerant network. *IEEE Geosci. Remote. Sens. Lett.* **19**, 1–5. 10.1109/LGRS.2022.3198088 (2022).

[19] Saleh, T., Weng, X., Holail, S., Hao, C. & Xia, G. S. Dam-net: Flood detection from sar imagery using differential attention metric-based vision transformers. *ISPRS J. Photogramm. Remote. Sens.* **212**, 440–453. 10.1016/J.ISPRSJPRS.2024.05.018 (2024).

[20] Tahermanesh, S., Mohammadzadeh, A., Mohsenifar, A. & Moghimi, A. Siscnet: A novel siamese inception-based network with spatial and channel attention for flood detection in sentinel-1 imagery. *Remote. Sens. Appl. Soc. Environ.* **38**, 101571. 10.1016/J.RSASE.2025.101571 (2025).

[21] Yu, Y. & Acton, S. Speckle reducing anisotropic diffusion. *IEEE Transactions on Image Process.* **11**, 1260–1270. 10.1109/TIP.2002.804276 (2002). 10.1109/TIP.2002.804276 18249696

[22] Li, H.-C., Celik, T., Longbotham, N. & Emery, W. J. Gabor feature based unsupervised change detection of multitemporal sar images based on two-level clustering. *IEEE Geosci. Remote. Sens. Lett* **12**, 2458–2462. 10.1109/LGRS.2015.2484220 (2015).

[23] Rao, Y., Zhao, W., Zhu, Z., Lu, J. & Zhou, J. Global filter networks for image classification (2021). arXiv:2107.00645.

[24] Vaswani, A. et al. Attention is all you need. *CoRR***abs/1706.03762** (2017). arXiv:1706.03762.

